# Recent trends in the prevalence of underweight, overweight, and obesity in Korean adults: The Korean National Health and Nutrition Examination Survey from 1998 to 2014

**DOI:** 10.1016/j.je.2016.08.014

**Published:** 2017-04-15

**Authors:** Hyun-Young Shin, Hee-Taik Kang

**Affiliations:** a Department of Family Medicine, Myongji Hospital, Seonam University, College of Medicine, Gyeonggi-do, Republic of Korea; b Department of Epidemiology and Health Promotion and Institute for Health Promotion, Graduate School of Public Health, Yonsei University, Seoul, Republic of Korea; c Department of Family Medicine, School of Medicine, Chungbuk National University, Cheongju-city, Republic of Korea

**Keywords:** Obesity, Overweight, Prevalence, Underweight, Visceral obesity

## Abstract

**Background:**

Recent obesity studies have reported that the rising trend in obesity has stabilized or leveled off. Our study aimed to update estimates of the recent prevalence trend in obesity based on the Korean National Health and Nutrition Examination Survey 1998–2014.

**Methods:**

A total of 66,663 subjects were included and defined as being either underweight, overweight, or obese, in accordance with a BMI of 18.5 kg/m^2^ or lower, 23 kg/m^2^ or higher, and 25 kg/m^2^ or higher, respectively.

**Results:**

The prevalence of underweight in KNHANES I through VI surveys was 5.4%, 6.1%, 5.8%, 6.5%, 7.6%, and 7.5%, respectively, in men (*p* for trend = 0.04, β = 0.003) and 4.7%, 3.3%, 3.4%, 3.3%, 2.7%, and 2.6%, respectively, in women (*p* for trend = 0.03, β = −0.002). Also for KNHANES I through VI, the respective prevalence of overweight/obesity was 50.3%, 57.2%, 62.5%, 62.3%, 61.4%, and 61.3% in men (*p* for trend<0.01, β = 0.009) and 48.3%, 50.3%, 50.0%, 47.8%, 47.0%, and 45.3% in women (*p* for trend<0.01, β = −0.01), respectively.

**Conclusions:**

The obesity occurrence in men was trending upward with respect to overweight/obesity and for grade 1 and 2 obesity, but not for abdominal obesity. However, the obesity trends in women were leveling off from overweight/obesity, grade 1 obesity, and abdominal obesity measures. Further studies are required with data on muscle mass and adiposity for effective obesity control policies.

## Highlights

• We conducted this study using the KNHANES from 1998 to 2014.
• Male obesity showed upward trends in overweight/obesity and grade 1 and 2 obesity.
• Overweight/obesity, grade 1, and abdominal obesity were leveling off in females.
• The prevalence of underweight decreased in men but increased in women.

## Introduction

Obesity is disease of global epidemic proportions that has increased during the recent decades.^1^ It is related to non-communicable diseases, such as type 2 diabetes, hypertension, dyslipidemia, cardiovascular diseases, musculoskeletal disorders, and certain malignancies,^2^ as these are associated with environmental factors, including family lifestyles and unhealthy behaviors. The World Health Organization (WHO) aims to stop obesity by 2025 as part of the program called “Global Action Plan for the Prevention and Control of Non-Communicable Diseases 2013–2020”,^3^ and has suggested diverse efforts, such as following a healthy diet with physical activity, for prevention. It has also advocated regular monitoring of epidemiological facts in obesity.

Recent studies have reported that the rising trends of obesity have stabilized or leveled off in some European countries and China.^4^ However, several Korean studies have reported that the trends of obesity in Korea have increased in men, but neither increased nor decreased in women.^5^, ^6^, ^7^ It should be noted that more recent trends still needs to be reported. Our study, therefore, aimed to report more recent trends in obesity prevalence in Korea by including a longer time frame of years, from 1998 through 2014, than previous reports and assessing various measures, including abdominal obesity and the population of underweight, based on data from the Korean National Health and Nutrition Examination Survey (KNHANES).

## Methods

The KNHANES is a cross-sectional, nationally representative survey conducted by the Korean Ministry of Health and Welfare and has been performed in 6 phases: the KNHANES phase I (1998), II (2001), III (2005), IV (2007–2009), V (2010–2012), and VI (2013–2015) studies. As the most recent data (from 2015) was unavailable, the data from 1998 to 2014 are included in this study. The KNHANES is composed of a Health Interview Survey, a Health Behavior Survey, a Health Examination Survey, and a Nutrition Survey. Households as sampling units were stratified, and the information was collected through a multistage probability-based sampling design based on sex, age, and geographic area, using household registries. On completion of this survey, participants provided written informed consent for use of their data for further analyses and were given the right to refuse to participate, in accordance with the National Health Enhancement Act.

Of the 177,056 participants in the KNHANES I-VI, we excluded those younger than 20 years and those who did not have information for body mass index (BMI) (n = 10,393). After these exclusions, a total of 66,663 participants (28,594 men and 38,069 women) were included in the final analysis. The Institutional Review Board (IRB) of the Korea Centers for Disease Control and Prevention approved this study.

### Measurement and definition of underweight, overweight, and obesity

Physical examinations were performed by a trained medical staff, following standardized procedures. Body weight, height, and waist circumference were measured to the nearest 0.1 kg and 0.1 cm, respectively, with participants wearing light indoor clothing without shoes. Waist circumference was checked at the midpoint between the lower border of the rib cage and the iliac crest. BMI was calculated as the ratio of weight in kilograms to height in meters squared (kg/m^2^). According to the Asia-Pacific regional guidelines of the WHO and International Obesity Task Force, we defined the cutoff points for being underweight, overweight, and obesity as a BMI of 18.5 kg/m^2^ or lower, 23 kg/m^2^ or higher, and 25 kg/m^2^ or higher, respectively.^8^ We further stratified obesity as grade 1 (BMI ≥25 kg/m^2^) and grade 2 (BMI ≥30 kg/m^2^), which were not mutually exclusive. Abdominal obesity was defined as waist circumference ≥90 cm for men and ≥85 cm for women using the cut-off points in Korea.^9^

### Statistical analysis

KNHANES data from the Korea National Statistical Office were used to define the standard population. In order to represent the entire Korean adults without biased estimates, sampling weights were applied to account for the complex sampling, which included stratification by district at the first step and stratification by sex and age at the second step. Furthermore, to avoid bias from changes in age and sex distributions in each phase, adjustments to age and sex were made using the Korean population distribution in the year 2005.

All data on continuous variables were presented as means (standard errors [SEs]) and one-way analysis of variance was used to compare mean values for continuous variables, such as age, BMI, and waist circumference, across KNHANES phases. Linear regression analyses for P trends and logistic regression analyses for odds ratios (ORs) were used. All analyses were conducted using SAS statistical software (version 9.2; SAS Institute Inc., Cary, NC, USA). All statistical tests were two-sided, and statistical significance was determined at *P*-value <0.05.

## Results

The 66,663 participants included in this study were distributed as follows: 7962 from KNHANES I, 6572 from KNHANES II, 5462 from KNHANES III, 17,126 from KNHANES IV, from 18,255 KNHANES V, and 11,286 from KNHANES VI (Table 1).

**Table 1:** Study subjects according to KNHANES phase, age group, and sex.

	Total	I (1998)	II (2001)	III (2005)	IV (2007–2009)	V (2010–2012)	VI (2013–2014)
**Men, unweighted *n* (%)**	28,594 (100)	3597 (100)	2864 (100)	2325 (100)	7254 (100)	7769 (100)	4785 (100)
20–39 years	9417 (32.9)	1582 (44.0)	1192 (41.6)	768 (33.0)	2354 (32.5)	2172 (28.0)	1349 (28.2)
40–59 years	10,933 (38.2)	1350 (37.5)	1125 (39.3)	1003 (43.1)	2720 (37.5)	2915 (37.5)	1820 (38.0)
≥60 years	8244 (28.8)	665 (18.5)	547 (19.1)	554 (23.8)	2180 (31.1)	2682 (34.5)	1616 (33.8)
**Women, unweighted *n* (%)**	38,069 (100)	4365 (100)	3708 (100)	3137 (100)	9872 (100)	10,486 (100)	6501 (100)
20–39 years	12,750 (33.5)	1879 (43.0)	1561 (42.1)	1128 (36.0)	3230 (32.7)	3108 (29.6)	1844 (28.4)
40–59 years	14,059 (36.9)	1540 (35.3)	1343 (36.2)	1248 (39.8)	3578 (36.2)	3883 (37.0)	2467 (37.9)
≥60 years	11,260 (29.6)	946 (21.7)	804 (21.7)	761 (24.3)	3064 (31.0)	3495 (33.3)	2190 (33.7)

Table 2 shows the changes in BMI and waist circumference according to sex and age group (20–39 years, 40–59 years, and 60 years or older) across the KNHANES phases. Mean BMI in men increased overall across the KNHANES phases (*p* for trend<0.001, β = 0.12), whereas it decreased in women (*p* for trend = 0.03, β = −0.05). Mean BMI increased in men of all age groups (all *p* for trend<0.05), whereas it only decreased in the middle-aged group among women (*p* for trend<0.001, β = −0.18). Waist circumference in men showed no significant change overall (*p* for trend = 0.65, β = 0.03), as waist circumference increased in the youngest group but decreased for the middle-aged group across the KNHANES phases (*p* for trend = 0.002, β = 0.30 and *p* for trend<0.001, β = −0.29, respectively). However, waist circumference in women decreased overall (*p* for trend<0.001, β = −0.24), as the waist circumference of the middle-aged and the oldest groups decreased across the KNHANES phases (*p* for trend<0.001, β = −0.60 and *p* for trend<0.001, β = −0.41, respectively).

**Table 2:** Weighted estimates of body mass index and waist circumference based on the KNHANES I–VI.

		I (1998)	II (2001)	III (2005)	IV (2007–2009)	V (2010–2012)	VI (2013–2014)	P value	β coefficient	*p* for trend
**Men**	Age, years	42.2 (0.32)	45.7 (0.38)	42.8 (0.44)	43.8 (0.25)	44.8 (0.28)	45.6 (0.32)	<0.01	0.30	0.01
BMI, kg/m^2^	All	23.2 (0.07)	23.7 (0.07)	24.0 (0.08)	24.0 (0.05)	24.1 (0.05)	24.2 (0.06)	<0.01	0.12	<0.01
	20–39 years	23.1 (0.10)	23.6 (0.11)	23.7 (0.14)	24.0 (0.08)	24.2 (0.10)	24.3 (0.11)	<0.01	0.17	<0.01
	40–59 years	23.7 (0.09)	24.1 (0.09)	24.4 (0.11)	24.4 (0.06)	24.3 (0.06)	24.6 (0.08)	<0.01	0.07	<0.01
	≥60 years	22.0 (0.13)	23.0 (0.17)	23.4 (0.18)	23.3 (0.09)	23.5 (0.08)	23.5 (0.08)	<0.01	0.12	<0.01
WC, cm	All	82.8 (0.20)	84.4 (0.19)	83.7 (0.23)	84.2 (0.15)	84.2 (0.16)	84.2 (0.16)	<0.01	0.03	0.65
	20–39 years	81.2 (0.28)	82.6 (0.29)	81.4 (0.37)	82.7 (0.23)	82.9 (0.27)	83.1 (0.29)	<0.01	0.30	<0.01
	40–59 years	85.1 (0.25)	86.0 (0.25)	86.0 (0.28)	85.4 (0.19)	85.0 (0.19)	85.0 (0.22)	<0.01	−0.29	<0.01
	≥60 years	82.2 (0.41)	85.0 (0.46)	85.1 (0.55)	85.0 (0.26)	85.2 (0.25)	84.8 (0.28)	<0.01	−0.04	0.74
**Women**	Age, years	43.3 (0.39)	45.6 (0.39)	44.6 (0.41)	45.9 (0.26)	47.0 (0.26)	47.8 (0.35)	<0.01	0.70	<0.01
BMI, kg/m^2^	All	23.1 (0.06)	23.4 (0.07)	23.3 (0.09)	23.2 (0.05)	23.3 (0.06)	23.2 (0.06)	<0.01	−0.05	0.03
	20–39 years	22.2 (0.08)	22.2 (0.09)	22.2 (0.12)	22.1 (0.08)	22.1 (0.09)	22.1 (0.10)	0.33	−0.03	0.32
	40–59 years	24.1 (0.09)	24.2 (0.10)	24.2 (0.12)	23.8 (0.06)	23.9 (0.07)	23.5 (0.08)	<0.01	−0.18	<0.01
	≥60 years	23.8 (0.12)	24.3 (0.16)	24.3 (0.14)	24.3 (0.08)	24.3 (0.08)	24.3 (0.09)	<0.01	−0.01	0.90
WC, cm	All	78.0 (0.21)	78.8 (0.23)	77.9 (0.26)	78.4 (0.18)	78.1 (0.17)	77.5 (0.20)	<0.01	−0.24	<0.01
	20–39 years	74.2 (0.23)	74.3 (0.25)	73.8 (0.34)	74.4 (0.23)	73.7 (0.25)	73.7 (0.28)	<0.01	−0.14	0.12
	40–59 years	80.7 (0.30)	80.3 (0.31)	79.8 (0.33)	79.6 (0.21)	79.3 (0.22)	77.6 (0.23)	<0.01	−0.60	<0.01
	≥60 years	83.2 (0.38)	84.5 (0.43)	83.4 (0.43)	83.8 (0.26)	83.2 (0.23)	82.6 (0.29)	<0.01	−0.41	<0.01

Values are reported as mean (standard error).

BMI, body mass index; WC, waist circumference.

**Fig. 1:**
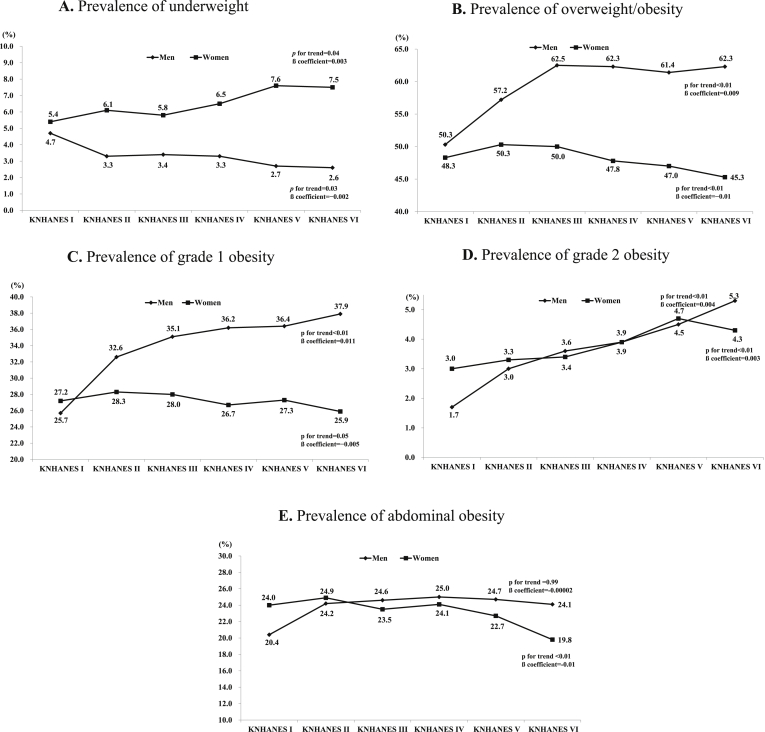
Prevalence of underweight, overweight/obesity, grade 1 obesity, grade 2 obesity, and abdominal obesity in men and women, according to KNHANES phase. 1A. Prevalence of being underweight. 1B. Prevalence of overweight/obesity. 1C. Prevalence of grade 1 obesity. 1D. Prevalence of grade 2 obesity. 1E. Prevalence of abdominal obesity. *P*-values for trends were determined by logistic regression analyses after setting the KNHANES phase as the continuous variable. Definition: Underweight, BMI <18.5 kg/m^2^; Overweight/obesity, BMI ≥23 kg/m^2^; Grade 1 obesity, BMI ≥25 kg/m^2^; Grade 2 obesity, BMI ≥30 kg/m^2^; Abdominal obesity, waist circumference ≥90 cm in men, ≥85 cm in women.

The prevalence of being underweight, overweight/obese, grade 1 and grade 2 obesity, or abdominal obesity is presented in Fig. 1. The prevalence of being underweight in KNHANES I through VI was 5.4%, 6.1%, 5.8%, 6.5%, 7.6%, and 7.5% in men (*p* for trend = 0.04, β = 0.003) and 4.7%, 3.3%, 3.4%, 3.3%, 2.7%, and 2.6% in women (*p* for trend = 0.03, β = −0.002). The underweight prevalence trend in men decreased with the KNHANES phase (*p* for trend = 0.002, β = −0.03), while that in women increased (*p* for trend = 0.04, β = 0.003). The KNHANES I through VI prevalence of overweight/obesity was 50.3%, 57.2%, 62.5%, 62.3%, 61.4%, and 61.3% in men (*p* for trend <0.01, β = 0.009) and 48.3%, 50.3%, 50.0%, 47.8%, 47.0%, and 45.3% in women (*p* for trend<0.01, β = −0.01), respectively. The prevalence trend of overweight/obesity in men increased (*p* for trend<0.01, β = 0.009), whereas that in women decreased (*p* for trend<0.01, β = −0.01). Trends in the prevalence of grade 1 obesity in both sexes were similar with that of overweight/obesity. The prevalence of grade 2 obesity in both sexes increased time-dependently (*p* for trend<0.01, β = 0.004 in men; *p* for trend<0.01, β = 0.003 in women), and recently, a decrease was observed in women alone. Also, the respective prevalence of abdominal obesity was 20.4%, 24.2%, 24.6%, 25.0%, 24.7%, and 24.1% in men (*p* for trend = 0.99, β = −0.00002) and 24.0%, 24.9%, 23.5%, 24.1%, 22.7%, and 19.8% in women (*p* for trend<0.01, β = −0.01). Male abdominal obesity showed no significant changes across the KNHANES phases, but female abdominal obesity showed a decrease (*p* for trend = 0.99, β = −0.00002 in men and *p* for trend<0.01, β = −0.01 in women).

Detailed information on the prevalence of underweight, overweight/obesity, grade 1 and 2 obesity, and abdominal obesity according to age group and sex is shown in Table 3. Prevalence of underweight decreased (*p* for trend<0.01, β = −0.01), but that of overweight/obesity increased (*p* for trend = 0.03, β = 0.015) for the oldest male group. The prevalence of grade 1 and 2 obesity and abdominal obesity increased in the youngest male group (*p* for trend<0.01, β = 0.018 for grade 1 obesity; *p* for trend<0.01, β = 0.008 for grade 2 obesity; and *p* for trend = 0.01, β = 0.01 for abdominal obesity). The prevalence of grade 2 obesity in the middle-aged men increased (*p* for trend = 0.03, β = 0.004), while that of abdominal obesity decreased (*p* for trend = 0.02, β = −0.01). The prevalence of being underweight in the youngest and middle-aged women increased (*p* for trend<0.01, β = 0.008 in the youngest women and *p* for trend<0.01, β = 0.005 in the middle-aged women), whereas that in the oldest women decreased (*p* for trend = 0.04, β = −0.004). Female overweight/obesity prevalence in the youngest and middle-aged group leveled off, especially for the KNHANES II group (*p* for trend = 0.02, β = −0.01 and *p* for trend<0.01, β = −0.03, respectively). However, the prevalence of grade 2 obesity tended to increase in the youngest women (*p* for trend<0.01, β = 0.005), but recently, a decreasing pattern was observed during the 2010–2012 year of the survey. There was decrease in the prevalence of abdominal obesity for the middle-aged and oldest women (*p* for trend<0.01, β = −0.02 and *p* for trend<0.01, β = −0.02, respectively).

**Table 3:** Prevalence of underweight, overweight/obesity, grade 1 obesity (BMI ≥25 kg/m^2^), grade 2 obesity (≥30 kg/m^2^), and abdominal obesity and based on the KNHANES I–VI.

		I (1998)	II (2001)	III (2005)	IV (2007–2009)	V (2010–2012)	VI (2013–2014)	P value	β coefficient	P for trend
**Men**										
Underweight	20–39 years	4.4 (0.7)	3.3 (0.6)	3.4 (0.7)	3.7 (0.5)	3.0 (0.4)	3.1 (0.5)	0.28	−0.0001	0.94
	40–59 years	2.3 (0.4)	1.6 (0.4)	2.0 (0.5)	2.0 (0.3)	1.7 (0.3)	1.8 (0.3)	0.29	−0.0003	0.80
	≥60 years	11.7 (1.4)	7.8 (1.4)	7.1 (1.2)	5.4 (0.6)	4.3 (0.5)	3.5 (0.4)	0.24	−0.01	<0.01
Overweight/Obesity	20–39 years	47.4 (1.6)	53.6 (1.6)	58.1 (2.0)	59.8 (1.1)	57.6 (1.2)	59.2 (1.2)	0.02	0.009	0.07
	40–59 years	60.5 (1.5)	65.1 (1.6)	70.0 (1.7)	68.2 (1.0)	67.7 (1.0)	67.9 (1.0)	<0.01	0.003	0.44
	≥60 years	33.9 (2.1)	48.5 (2.8)	57.0 (2.9)	55.1 (1.4)	56.9 (1.3)	57.9 (1.2)	<0.01	0.015	0.03
Grade 1 Obesity	20–39 years	24.4 (1.2)	31.5 (1.7)	31.9 (1.9)	35.0 (1.0)	35.3 (1.1)	38.1 (1.3)	<0.01	0.018	<0.01
	40–59 years	31.4 (1.6)	36.5 (1.6)	41.1 (1.9)	41.1 (1.0)	39.9 (1.1)	40.7 (1.0)	<0.01	0.006	0.20
	≥60 years	15.7 (1.6)	26.1 (2.5)	29.7 (2.7)	27.6 (1.2)	30.9 (1.2)	30.1 (1.1)	0.87	0.007	0.23
Grade 2 Obesity	20–39 years	2.2 (0.4)	3.6 (0.7)	4.7 (0.9)	5.5 (0.5)	6.7 (0.7)	7.1 (0.6)	<0.01	0.008	<0.01
	40–59 years	1.6 (0.3)	2.6 (0.5)	3.2 (0.7)	3.0 (0.4)	3.0 (0.4)	4.4 (0.5)	<0.01	0.004	0.03
	≥60 years	0.1 (0.1)	2.1 (0.9)	1.2 (0.4)	1.7 (0.3)	1.3 (0.3)	1.8 (0.4)	<0.01	0.000003	1.00
Abdominal Obesity	20–39 years	14.6 (1.1)	19.0 (1.3)	16.8 (1.5)	20.1 (0.9)	21.5 (1.0)	21.8 (1.0)	<0.01	0.01	0.01
	40–59 years	27.7 (1.4)	28.8 (1.5)	30.9 (1.5)	29.0 (1.0)	26.6 (1.0)	25.6 (1.0)	<0.01	−0.01	0.02
	≥60 years	20.0 (1.9)	28.8 (2.7)	33.3 (2.8)	30.7 (1.2)	30.3 (1.2)	27.6 (1.1)	0.02	−0.007	0.30
**Women**										
Underweight	20–39 years	8.0 (0.7)	9.7 (0.8)	10.5 (1.2)	11.8 (0.7)	13.9 (0.8)	13.1 (0.7)	<0.01	0.008	<0.01
	40–59 years	1.8 (0.4)	1.7 (0.4)	1.2 (0.3)	2.4 (0.3)	2.8 (90.3)	3.5 (0.4)	<0.01	0.005	<0.01
	≥60 years	6.4 (0.9)	4.5 (0.8)	4.1 (0.9)	2.7 (0.3)	2.9 (0.3)	2.3 (0.3)	<0.01	−0.004	0.04
Overweight/Obesity	20–39 years	33.6 (1.3)	34.2 (1.3)	32.6 (1.6)	31.7 (1.0)	32.1 (1.1)	31.3 (1.1)	<0.01	−0.01	0.02
	40–59 years	60.1 (1.5)	62.6 (1.6)	61.6 (1.8)	56.8 (1.0)	55.5 (1.0)	51.5 (1.0)	<0.01	−0.03	<0.01
	≥60 years	58.9 (1.8)	62.7 (2.2)	66.6 (2.1)	66.2 (1.1)	64.0 (1.1)	64.4 (1.1)	<0.01	−0.003	0.61
Grade 1 Obesity	20–39 years	17.3 (1.0)	16.0 (1.0)	16.6 (1.4)	16.4 (0.8)	18.3 (0.9)	17.4 (0.9)	<0.01	0.002	0.48
	40–59 years	35.0 (1.4)	36.3 (1.6)	34.5 (1.7)	31.2 (0.9)	31.6 (0.9)	28.7 (0.9)	0.03	−0.02	<0.01
	≥60 years	34.4 (1.8)	40.4 (2.1)	40.9 (2.0)	41.2 (1.1)	39.2 (1.1)	39.1 (1.0)	<0.01	−0.007	0.19
Grade 2 Obesity	20–39 years	2.2 (0.4)	1.8 (0.3)	2.7 (0.5)	3.2 (0.4)	4.3 (0.5)	3.8 (0.4)	0.04	0.005	<0.01
	40–59 years	3.5 (0.5)	3.9 (0.6)	4.7 (0.8)	4.3 (0.4)	4.9 (0.5)	4.2 (0.4)	<0.01	0001	0.94
	≥60 years	4.1 (0.6)	5.4 (1.0)	2.7 (0.6)	4.7 (0.5)	5.4 (0.5)	5.8 (0.5)	0.73	0.003	0.25
Abdominal Obesity	20–39 years	11.8 (0.8)	11.8 (1.0)	11.3 (1.2)	12.8 (0.7)	12.7 (0.8)	12.0 (0.8)	<0.01	0.00005	0.99
	40–59 years	29.1 (1.4)	27.8 (1.5)	26.3 (1.6)	25.8 (0.9)	23.5 (0.9)	18.5 (0.8)	0.03	−0.02	<0.01
	≥60 years	41.1 (1.9)	47.9 (2.2)	45.0 (2.1)	45.7 (1.3)	43.0 (1.2)	39.3 (1.1)	<0.01	−0.02	<0.01

Values are reported as mean (standard error).

**Fig. 2:**
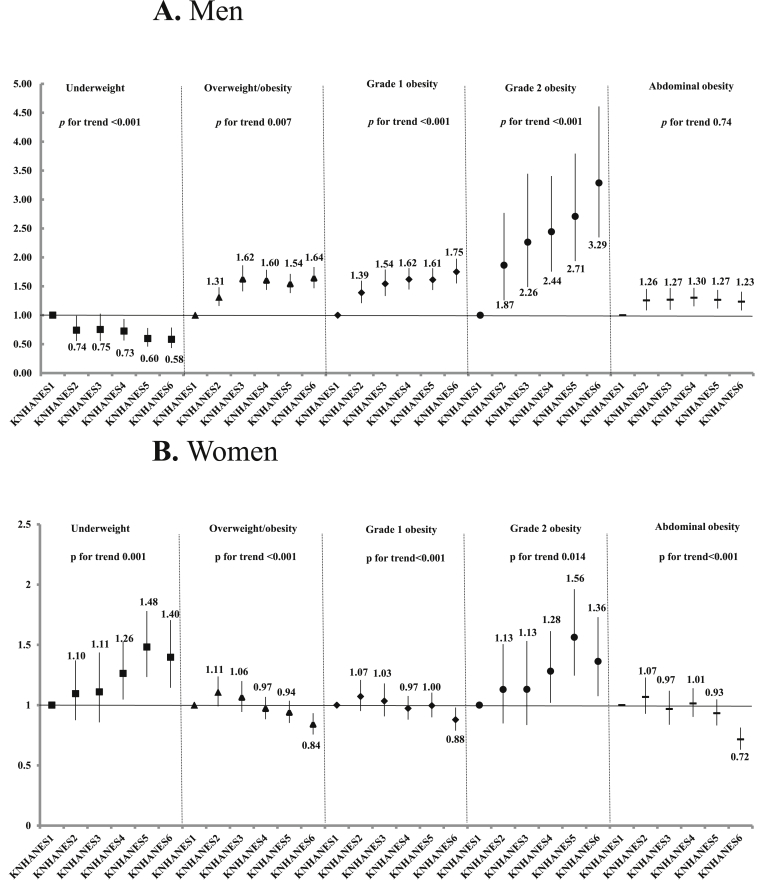
Odds ratios estimated by adjusting age groups (20–39 years, 40–59 years, and ≥60 years) for the prevalence of being underweight, having overweight/obesity, grade 1 obesity, grade 2 obesity and abdominal obesity in men and women, according to the KNHANES phase. 2A. Men. 2B. Women. *P*-values for trends were determined by logistic regression analyses after setting KNHANES phase as the continuous variable. Definition: Underweight, BMI <18.5 kg/m^2^; Overweight/obesity, BMI ≥23 kg/m^2^; Grade 1 obesity, BMI ≥25 kg/m^2^; Grade 2 obesity, BMI ≥30 kg/m^2^; Abdominal obesity, waist circumference ≥90 cm in men, ≥85 cm in women.

The trends in underweight, overweight/obese, grade 1 and 2 obesity, and abdominal obesity during the 1998–2014 survey are presented as ORs (95% CIs), and these indicated an estimated increase in the odds for obesity occurrence after adjusting for age group (Fig. 2). Compared with KNHANES I, the ORs for male prevalence of overweight/obesity, and grade 1 and 2 obesity in KNHANES II to VI was statistically significant and increased in a time-dependent manner (*p* for trend = 0.007, *p* < 0.001, and *p* < 0.001, respectively), while those for being underweight were significantly decreased (*p* for trend<0.001). In contrast, the odds for being underweight in women were statistically higher in KNHANES II–VI than in KNHANES I and tended to increase with the KNHANES phases (*p* for trend = 0.0001). Women showed a tendency of decreased prevalence of overweight/obesity, grade 1 obesity, and abdominal obesity (all *p* for trend<0.001), but an increased prevalence in grade 2 obesity (*p* for trend = 0.014).

## Discussion

Analyses of KNHANES data for 1998–2014 showed sex differences in trends in underweight, overweight/obesity, grade 1 and 2 obesity, and abdominal obesity prevalence among the Korean adults. The prevalence of underweight in men decreased, but that in women increased with the KNHANES phases. It is of note that the association between underweight and health outcomes is still controversial. Jee et al reported that underweight men and women had higher mortality;^10^ however, a recent Korean study found that underweight was not associated with increased mortality compared to a normal-weight population.^11^

From the KNHANES data, the trends in overweight/obesity and grade 1 and 2 obesity were in an upward trajectory in men. In contrast, the female trends were downward pointing in overweight/obesity and grade 1 obesity categories, and also recently decreasing in grade 2 obesity. There was a decrease in prevalence of abdominal obesity in women, but no significant change was seen in men.

We compared our present results with those of similar previous studies. Kang et al reported an upward trend of overweight/obesity and obesity in men and a slightly decreasing trend of overweight/obesity in women.^5^ Khang et al reported that obesity and abdominal obesity were increased in men and old women, but not in women of other ages.^6^ Yoo et al reported that obesity and abdominal obesity were increased in men, but not in women.^7^ Flegal's obesity study in the United States from 1999 to 2008 reported that an increasing trend was not observed in men and women;^12^ however, a recent report from 2005 to 2014 showed an increasing trend in overall and class 3 obesity in women and no change in men.^13^

These changes in the obesity epidemic in Korea might be closely related to changes in healthy behavior, although the direction of change is controversial. According to the Korea Health Statistics, which is published by the Korea Centers for Disease Control and Prevention, the trend of daily intake calorie for years 1998, 2001, 2005, 2007, 2008, 2009, 2010, 2011, 2012, and 2013 year were as follows: 2152 kcal, 2107 kcal, 2214 kcal, 2107 kcal, 2146 kcal, 2391 kcal, 2360 kcal, 2310 kcal, and 2386 kcal, respectively, for men, and 1729 kcal, 1713 kcal, 1742 kcal, and 1549 kcal, 1583 kcal, 1590 kcal, 1733 kcal, 1699 kcal, 1682 kcal, and 1779 kcal, respectively, for women.^14^ A trend of increasing daily caloric intake was observed in men, and a fluctuation in daily intake according to year was observed in women. In Kang et al's study, an increase in regular exercise and a decrease in regular alcohol consumption were observed in men, and active management of chronic disease was increased in both men and women from 1998 to 2009.^5^ Shin et al's study showed that the levels of exercise in men had greatly increased compared to women from 1998 to 2009.^15^ Kang et al's study reported an increased risk of alcohol use and marginally decreased walking rate from the 2008–2014 Korea Community Health Survey.^16^

Interestingly, our study shows that the rate of obesity has increased overall, but that for abdominal obesity has reached a plateau in men. This discrepancy can be explained by an increase in BMI in men, which is not due to an increase in adiposity but from an increase in muscle mass. Several studies reported that the exercise rate in men has increased.^5^, ^15^ The Korean government has emphasized a reduction in abdominal obesity via a multimedia campaign and development of new health policy. These efforts also help in screening for obesity-related diseases and consultation for risk factors for the individuals by providing free health check-ups. Furthermore, with the development of information technology and wider use of smart phones in Korea, applications and internet-based material about healthy behavior and building an attractive appearance have become popular among the larger population. These environmental changes could have influenced the popular view that a muscular body shape in men and a thin body shape in women are desirable in Korea. For these reasons, not only an increase in caloric intake but also an increase in muscle mass resulting from an increase in the exercise rate might be co-contributors to an increase in BMI in men. As an example, to spend the increased intake of food calories of about 200 kcal — as seen between 1998 and 2013–54 min of additional exercise, such as walking at 5 km/h (3.2 metabolic equivalent), is required by a 70 kg-weighted male. In men who exceed the average daily caloric intake between 1998 and 2013, incremental exercise would not be sufficient to offset the accumulation of extra calories as fat.^17^ In our study, although there were no definite changes in the trends of daily caloric intake and exercise rate for women, the discrepancy between the obese and underweight was increased, with observed gradual increases both in grade 2 obesity and underweight. From surveys and behavioral studies, it has been reported that both a negative stereotype of obese people and distorted body image bring about extreme self-control for weight loss and an increase in the underweight population among Korean women,^18^, ^19^ and further studies on trends for changes in average muscle mass and adiposity are needed in the future.

Our study has several limitations that should be considered when interpreting the results. First, this was a cross-sectional study, which could not identify cause-and-effect relationships. Second, those who had missing anthropometric data were excluded in this study, so selection bias could not be excluded, as overweight or obese women tend to avoid anthropometric measurements. Third, overweight and obesity were defined based on BMI numbers, which is calculated from body weight and height. Thus, we cannot distinguish whether these trends resulted from body fat or muscle mass changes or both. Therefore, misclassified adiposity may lead to overestimation of being fat in muscular participants, as BMI is strongly correlated with both muscle and fat mass.^20^ Finally, parameters of obesity, caloric intake, and physical activity were not measured over the same relevant phase. For instance, because the Korea Centers for Disease Control and Prevention did not comprehensively survey moderate- and vigorous-intensity physical activity before KNHANES III, we could not estimate the percentage of individuals who were regularly engaged in physical activity from KNHANES I to VI. These limitations make it difficult to identify conclusive cause-effect relationships.

Despite its potential limitations, this study has several strengths. First, to represent the general Korean population aged 20 years or older, we applied sampling weights to all analyses, and adjustment for age and sex was applied to the Korean population distribution in the year 2005. Second, we showed the prevalence trends in abdominal obesity based on waist circumference, in addition to measures of underweight, overweight/obese, and extent of obesity, as calculated by BMI. Thus, detailed information about the obesity pattern was gathered. Third, this is the most recent study on Korean obesity and the first to include data from 2014.

In summary, the trend in obesity prevalence increased without a change in abdominal obesity in men, and overweight/obesity and abdominal obesity prevalence decreased in women during the 17-year period.

Further studies on trends of muscle mass and adiposity are needed for establishment of effective obesity control policies through targeted interventions.

## Conflicts of interest

None declared.

## References

[1] Ng M., Fleming T., Robinson M. (2014). Global, regional, and national prevalence of overweight and obesity in children and adults during 1980-2013: a systematic analysis for the Global Burden of Disease Study 2013. Lancet.

[2] World health organization. Overweight and obesity statistics, Accessed at http://www.who.int/mediacentre/factsheets/fs311/en/ (March 30, 2016).

[3] World Health Assembly (2013). Follow-up to the Political Declaration of the High-level Meeting of the General Assembly on the Prevention and Control of Non-communicable Diseases. http://apps.who.int/gb/ebwha/pdf_files/WHA66/A66_R10-en.pdf.

[4] Rokholm B., Baker J.L., Sørensen T.I. (2010). The levelling off of the obesity epidemic since the year 1999 – a review of evidence and perspectives. Obes Rev.

[5] Kang H.T., Shim J.Y., Lee H.R., Park B.J., Linton J.A., Lee Y.J. (2014). Trends in prevalence of overweight and obesity in Korean adults, 1998–2009: the Korean National Health and Nutrition Examination Survey. J Epidemiol.

[6] Khang Y.H., Yun S.C. (2010). Trends in general and abdominal obesity among Korean adults: findings from 1998, 2001, 2005, and 2007 Korea National Health and Nutrition Examination Surveys. J Korean Med Sci.

[7] Yoo S., Cho H.J., Khang Y.H. (2010). General and abdominal obesity in South Korea, 1998–2007: gender and socioeconomic differences. Prev Med.

[8] International Obesity Task Force & World Health Organization (2000). The Asian-Pacific Perspective: Redefining Obesity and its Treatment. http://www.wpro.who.int/nutrition/documents/Redefining_obesity/en/index.html.

[9] Lee S.Y., Park H.S., Kim D.J. (2007). Appropriate waist circumference cutoff points for central obesity in Korean adults. Diab Res Clin Pract.

[10] Jee S.H., Sull J.W., Park J. (2006). Body-mass index and mortality in Korean men and women. N Engl J Med.

[11] Lee J.Y., Kim H.C., Kim C. (2016). Underweight and mortality. Public Health Nutr.

[12] Flegal K.M., Carroll M.D., Ogden C.L., Curtin L.R. (2010). Prevalence and trends in obesity among US adults, 1999-2008. JAMA.

[13] Flegal K.M., Kruszon-Moran D., Carroll M.D., Fryar C.D., Ogden C.L. (2016). Trends in obesity among adults in the United States, 2005 to 2014. JAMA.

[14] Korea Centers for Disease Control and Prevention. Korea Health Statistics 2014: Korea National Health and Nutrition Examination Survey (KNHANES VI). Accessed at https://knhanes.cdc.go.kr/knhanes/eng/index.do (March 30, 2016).

[15] Shin J.H., Dupre M.E., Østbye T., Murphy G., Silberberg M. (2015). The relationship of socioeconomic and behavioral risk factors with trends of overweight in Korea. J Prev Med Public Health.

[16] Kang Y.W., Ko Y.S., Kim K.Y., Sung C., Lee D.H., Jeong E. (2015). Trends in health-related behaviors of Korean adults: study based on data from the 2008-2014 Community Health Surveys. Epidemiol Health.

[17] Jetté M., Sidney K., Blümchen G. (1990). Metabolic equivalents (METS) in exercise testing, exercise prescription, and evaluation of functional capacity. Clin Cardiol.

[18] Kinzl J.F. (2016). Obesity: stigmatization, discrimination, body image. Wien Med Wochenschr.

[19] Choi O.J., Cho Y.G., Kang J.H. (2013). Weight control attempts in underweight Korean adults: Korea national health and nutrition examination survey, 2007-2010. Korean J Fam Med.

[20] Gallagher D., Visser M., Sepúlveda D., Pierson R.N., Harris T., Heymsfield S.B. (1996). How useful is body mass index for comparison of body fatness across age, sex, and ethnic groups?. Am J Epidemiol.

