# Recent Trends in the Prevalence of Underweight, Overweight, and Obesity in Korean Adults: Korean National Health and Nutrition Examination Survey From 1998 to 2013

**DOI:** 10.2188/jea.JE20170106

**Published:** 2018-02-05

**Authors:** Hyun-Young Shin, Hee-Taik Kang

**Affiliations:** 1 Department of Family Medicine, Myongji Hospital, Seonam University, College of Medicine, Gyeonggi-do, Republic of Korea; 2 Department of Epidemiology and Health Promotion and Institute for Health Promotion, Graduate School of Public Health, Yonsei University, Seoul, Republic of Korea; 3 Department of Family Medicine, School of Medicine, Chungbuk National University, Cheongju-city, Republic of Korea

There were several numerical errors in this article, and we have corrected data in the abstract and results sections. First, in the results section of the abstract (page 413, 1st line), the prevalence rates of underweight in men and women were reversed and should read as follows: “4.7%, 3.3%, 3.4%, 3.3%, 2.7%, and 2.6%, respectively, in men (*P* for trend = 0.03, β = −0.002) and 5.4%, 6.1%, 5.8%, 6.5%, 7.6%, and 7.5%, respectively, in women (*P* for trend = 0.04, β = 0.003).”

Second, in the results section of the abstract (page 413, 4th line), the prevalence of overweight/obesity in KNHANES VI should be amended as “62.3%” in men.

Third, in the results section of the main text (page 414, 4th line of the 3rd paragraph), the prevalence rates of underweight in men and women were reversed and should read as follows: “4.7%, 3.3%, 3.4%, 3.3%, 2.7%, and 2.6%, respectively, in men (*P* for trend = 0.03, β = −0.002) and 5.4%, 6.1%, 5.8%, 6.5%, 7.6%, and 7.5%, respectively, in women (*P* for trend = 0.04, β = 0.003).”

Fourth, in the results section of the main text (page 414, 10th line of the 3rd paragraph), the prevalence of overweight/obesity in KNHANES VI should be amended as “62.3%” in men.

Fifth, in the result section of main text (page 415, 13th line of the 5th paragraph), *P* for trend of the odds for being underweight in women should read as follows: *P* for trend = 0.001. The authors apologize for these errors.

